# Paradoxical proepileptic response to NMDA receptor blockade linked to cortical interneuron defect in *stargazer* mice

**DOI:** 10.3389/fncel.2013.00156

**Published:** 2013-09-18

**Authors:** Atul Maheshwari, Walter K. Nahm, Jeffrey L. Noebels

**Affiliations:** ^1^Developmental Neurogenetics Laboratory, Department of Neurology, Baylor College of MedicineHouston, TX, USA; ^2^University of California, San Diego School of Medicine, San DiegoCA, USA; ^3^Developmental Neurogenetics Laboratory, Department of Neuroscience, Baylor College of MedicineHouston, TX, USA; ^4^Developmental Neurogenetics Laboratory, Department of Molecular and Human Genetics, Baylor College of MedicineHouston, TX, USA

**Keywords:** absence epilepsy, stargazin, parvalbumin, cortical interneurons, GluA4, disinhibition

## Abstract

Paradoxical seizure exacerbation by anti-epileptic medication is a well-known clinical phenomenon in epilepsy, but the cellular mechanisms remain unclear. One possibility is enhanced network disinhibition by unintended suppression of inhibitory interneurons. We investigated this hypothesis in the *stargazer *mouse model of absence epilepsy, which bears a mutation in stargazin, an AMPA receptor trafficking protein. If AMPA signaling onto inhibitory GABAergic neurons is impaired, their activation by glutamate depends critically upon NMDA receptors. Indeed, we find that *stargazer *seizures are exacerbated by NMDA receptor blockade with CPP (3-[(R)-2-carboxypiperazin-4-yl]-prop-2-enyl-1-phosphonic acid) and MK-801, whereas other genetic absence epilepsy models are sensitive to these antagonists. To determine how an AMPA receptor trafficking defect could lead to paradoxical network activation, we analyzed stargazin and AMPA receptor localization and found that stargazin is detected exclusively in parvalbumin-positive (PV ^+^) fast-spiking interneurons in somatosensory cortex, where it is co-expressed with the AMPA receptor subunit GluA4. PV ^+^ cortical interneurons in *stargazer* show a near twofold decrease in the dendrite:soma GluA4 expression ratio compared to wild-type (WT) littermates. We explored the functional consequence of this trafficking defect on network excitability in neocortical slices. Both NMDA receptor antagonists suppressed 0 Mg ^2^^+^-induced network discharges in WT but augmented bursting in *stargazer* cortex. Interneurons mediate this paradoxical response, since the difference between genotypes was masked by GABA receptor blockade. Our findings provide a cellular locus for AMPA receptor-dependent signaling defects in *stargazer* cortex and define an interneuron-dependent mechanism for paradoxical seizure exacerbation in absence epilepsy.

## INTRODUCTION

The ability of an anti-epileptic drug (AED) to aggravate seizures is an unwelcome clinical problem affecting a small percentage of individuals with epilepsy (Gayatri and Livingston, 2006). However, due to the underlying heterogeneity of human seizure disorders, no common predictive biomarker or mechanism linked to this malignant form of pharmacoresistance has been identified. Paradoxical seizure enhancement by drugs that ordinarily reduce membrane depolarization or excitatory synaptic transmission suggests an innate difference in intrinsic cellular excitability or functional connectivity mediating the aberrant neuronal synchronization. One possibility, a pre-existing defect in the strength of inhibitory interneurons, may offer a specific mechanism for this idiosyncratic response. For example, phenytoin aggravates myoclonic seizures in a severe form of infantile epilepsy, Dravet syndrome, arising from mutation of *Scn1a* (Guerrini, 2012). Recent analysis of haploinsufficient *Scn1a* mouse mutants found a decreased density of inward sodium current that preferentially impaired high frequency discharges in interneurons (Yu et al., 2006; Ogiwara et al., 2007; Dutton et al., 2012). Further depression of their excitability by sodium channel blocking drugs such as phenytoin could synaptically disinhibit pyramidal cells, despite phenytoin’s simultaneous membrane suppressant effects on them. This example of malignant disinhibition arising from an inherently weakened interneuron population identifies a general mechanism for seizure exacerbation by otherwise potent anti-convulsant drugs.

In absence epilepsy, aggravation of seizures with anti-epileptic medication in some individuals is also well-described (Chaves and Sander, 2005; Thomas et al., 2006). In particular, GABAergic agents such as clonazepam, tiagabine, and vigabatrin that effectively terminate convulsive seizures typically provoke or prolong cortical spike-wave seizure patterns. An increase in tonic inhibition has been proposed (Crunelli et al., 2011) to explain the effects of these agents on thalamocortical oscillations in genetic models of epilepsy; however, the exact mechanism of seizure exacerbation with other anti-epileptic drugs lacking this property, such as lamotrigine, levetiracetam, and carbamazepine, is unknown (Nakken et al., 2003; Gayatri and Livingston, 2006). We identified a glutamate-related paradoxical response in the *stargazer* mouse model of absence epilepsy, where seizures are markedly exacerbated by 3-[(R)-2-carboxypiperazin-4-yl]-prop-2-enyl-1-phosphonic acid (CPP), a competitive NMDA receptor antagonist (Nahm and Noebels, 1998), which has also been noted to aggravate seizures in some patients with epilepsy (Sveinbjornsdottir et al., 1993). The *stargazer* phenotype of absence seizures and ataxia arises from mutation of *Cacng2*, which encodes the protein stargazin (Letts et al., 1998; Kato et al., 2010). Despite its gene symbol, this protein functions as a transmembrane AMPA receptor regulatory protein (TARP) critical for AMPA subunit clustering at synaptic and extrasynaptic sites in cerebellar granule cells (Chen et al., 2000). Although this explains the ataxic phenotype, the AMPA receptor trafficking deficit should also affect the thalamocortical loop implicated in the generation of absence seizures (Pinault, 2003; Meeren et al., 2005; Beenhakker and Huguenard, 2009). Each limb of the thalamocortical network is regulated locally through inhibitory GABAergic neurons in the neocortex and the nucleus of the reticular thalamus (RTN). A reduction of functional AMPA receptor current has been identified in *stargazer* RTN neurons (Menuz and Nicoll, 2008; Barad et al., 2012), and recently, a deficit in AMPA receptor expression has been reported in *stargazer *cortical interneurons *in vitro* (Tao et al., 2013). However, the exact cellular localization of stargazin in cortical interneuronal subpopulations and its contribution to AMPA receptor trafficking, cortical network excitability and the phenomenon of paradoxical AED response have not been explored.

Here we interrogated the cortical node of the thalamocortical loop, and demonstrate that in mouse somatosensory cortex, stargazin is exclusively expressed in PV^+^ cells, and that lack of functional stargazin reduces dendritic GluA4 trafficking in these interneurons. We also find that NMDA receptors play a key role in limiting abnormal cortical synchronization in *stargazer* mice, since both competitive and non-competitive antagonists lead to paradoxical seizure exacerbation *in vivo*. Interestingly, the response is specific to this gene mutation, since analogous seizures in *tottering *mice were appropriately blocked by NMDA receptor blockade. *In vitro*, we determined that cellular hyperexcitability differences unmasked by NMDA receptor blockade in Mg^2^^+^ deficient solution are abolished by GABA receptor blockade, isolating the *stargazer* cortical network excitability defect to interneurons.

## MATERIALS AND METHODS

### MICE

Experiments used adult homozygous *stargazer *mutants on a C57BL6/J background (*stg/stg*) and their wild-type (WT) littermate controls (+/+). To selectively label PV^+^ cells in WT**mice *in vivo*, we used a PV-Cre Ai9 (stop-floxed tdTomato) transgenic mouse line (Jackson Laboratory Stock #008069 and #007909). These mice have a >90% C57BL6/J background, with minimal contribution from the 129P3/J strain after four generations of cross-breeding, ultimately expressing Cre recombinase from the endogenous parvalbumin (*Pvalb)* locus (Madisen et al., 2010). However, due to the tight linkage of the *stg* and *Pvalb* loci on chromosome 15, we were unable to create a transgenic *stg* mutant expressing a *Pvalb*-driven reporter. Genotypes were confirmed by PCR of tail DNA with the following primers: GAGCAAGCAGGTTTCAGGC, TACTTCATCCGCCATCCTTC, and TGGCTTTCACTGTCTGTTGC, which produce a WT (360 bp) and mutant (155 bp) band (Burgess et al., 1997). All animal research was performed in accordance with Baylor College of Medicine Institutional Animal Care and Use Committee (IACUC) guidelines and regulations.

### *IN VIVO* VIDEO-EEG MONITORING

Mice were anesthetized by Avertin and surgically implanted with bilateral silver wire electrodes (0.005^″^ diameter) inserted into the subdural space over the parietal cortex bilaterally through cranial burr holes and attached to a microminiature connector cemented to the skull. Mice were allowed to recover for at least 48 h before analysis. EEG and behavioral activity in freely moving mice were analyzed using simultaneous video-EEG monitoring (Harmonie software version 6.1c, Stellate Systems). All EEG signals were filtered using a 0.3 Hz high-pass filter, 70 Hz low-pass filter, and 60 Hz notch filter. All *in vivo* experiments were initiated between 12 and 1 pm to prevent confounding diurnal variation. Mice were allowed to acclimate to the recording environment for 30 min, and video-EEG was then collected for a 30 min baseline sampling period, followed by intraperitoneal drug injection with either MK-801 (Sigma) or phosphate-buffered saline (PBS) and monitored for 3 h. Seizure activity, defined by spike and wave discharges with an amplitude greater than or equal to 2× baseline voltage with a corresponding video-recorded behavioral arrest, was quantified by visual inspection. Total seconds of seizure activity, independent of seizure frequency or duration, were counted and divided by the baseline seizure duration at 30 min epochs to create a ratio relative to the 30 min baseline sampling period. Statistical differences were tested using a repeated measures ANOVA with Bonferroni post-tests to compare groups over time (Prism 5, version 5.0d, GraphPad, CA, USA). Statistical significance was set at *p *< 0.05.

### IMMUNOCYTOCHEMISTRY

Adult, 6–7-week-old mice of either sex were anesthetized with isoflurane and perfused with PBS followed by 4% paraformaldehyde (PFA). Brains were extracted and post-fixed in PFA for 1 h, then soaked in 30% sucrose in PBS at 4°C overnight. Primary antibodies used for immunohistochemistry included: mouse anti-parvalbumin (Sigma, 1:1000 dilution) for co-labeling with rabbit anti-GluA4 (Millipore, 3 mg/ml); mouse anti-stargazin (Neuromab, 1:10 dilution) for co-labeling with rabbit anti-parvalbumin (Novus Biologicals), and rabbit anti-GluA4 as above. Secondary antibodies for immunofluorescence included: Alexa Fluor 488 F(ab′)_2_ fragment of goat anti-mouse IgG (H+L) 2 mg/ml and Alexa Fluor 555 F(ab′)_2_ fragment of goat anti-rabbit IgG (H+L) 2 mg/ml; both at 1:1000 dilution. The fixed brain was embedded in optimal cutting temperature (OCT) medium, mounted in a Cryostat and cut in 30 μm coronal sections. Slides were thawed at room temperature for 30 min, washed three times in PBS, blocked for 1 h in a 10% bovine serum albumin (BSA) and 0.3% Triton blocking solution in PBS, and then washed three times in blocking solution and incubated overnight at room temperature with primary antibodies. After washing in blocking solution three more times, slides were incubated with secondary fluorescent antibodies, then washed once in blocking solution and twice in PBS before mounting with ProLong Antifade medium. Investigators were blinded to the genotypes prior to visualization and analysis.

### CONFOCAL MICROSCOPY

Images were acquired with a Zeiss LSM 510 confocal microscope and analyzed with ImageJ software (NIH), using the same settings for both WT and mutant mice. For compartmental densitometric analysis of GluA4 subunit staining, somatosensory cortex was identified along the suprathalamic convexity in coronal slices where PV^+^/GluA4^+^ cells were identified at 63× magnification. Z-stacks through the cell body of PV^+^ cortical cells were obtained and then collapsed into two dimensions using the same transparency settings for both genotypes. A 10 × 2 μm^2^ rectangular region of interest was used to measure the pixel density of GluA4 staining along the axis of the longest dendrite, and a circular region of interest with a diameter of 5 μm in the somatic region at the base of this dendrite was similarly used to measure the pixel density in the soma. To define a reproducible and valid sample set for dendritic expression analysis, only PV^+^ stained cells with a soma greater than 10 μm in greatest diameter were accepted, and any cell where the longest primary dendrite had a length less than 10 μm or which branched within the first 10 μm was excluded from the analysis. The densitometric ratio between dendritic and somatic GluA4 was then determined for each PV^+^/GluA4^+^ neuron. Statistical differences were tested using the unpaired Student’s *t*-test (Prism 5, version 5.0d, GraphPad, CA, USA). Statistical significance was set at *p *< 0.05.

### ELECTROPHYSIOLOGY

Animals were sacrificed by cervical dislocation. Each brain was quickly removed and placed in cold (4°C) cutting saline (in mM: NaCl, 60; KCl, 3; NaH_2_PO_4_, 1.25; NaHCO_3_, 28; CaCl_2_, 0.5; MgCl_2_, 7; L-ascorbic acid, 0.6; sucrose, 110; and alpha-D(+)-glucose, 5; pH 7.4) saturated with 95% O_2_ and 5% CO_2_ (carbogen). Two coronal cuts were made to the whole brain in order to obtain a 0.4 cm block of tissue [including coronal sections 160–360 (Sidman, 1971)] containing parieto-temporal cortex. This freshly exposed surface of the block was affixed to a vibratome stage with cyanoacrylate glue, bathed with cold carbogenated cutting saline, and serial 350–400 μm brain slices were cut and placed in room temperature carbogenated aCSF (in mM: NaCl, 124; KH_2_PO_4_, 2; MgSO_4_, 1.25; CaCl_2_, 2; NaHCO_3_, 25; KCl, 2; and alpha-D(+)-glucose, 11; pH 7.4) for at least 60 min. Slices [coronal sections 280–320 (Sidman, 1971)] were then transferred to a humidified carbogen interface recording chamber and continuously perfused (100 ml/h) with carbogenated aCSF or 0 Mg^2^^+^ aCSF (normal aCSF, with MgSO_4_ omitted) maintained at 32°C. Slices were allowed to equilibrate in the chamber for 30 min before recordings were commenced.

Drugs were applied through a multiple 3-way valve perfusion system. The flow rate through the system was held constant (100 ml/h), and the volume of the bath was maintained at 0.5 ml (in the interface chamber) and 1.2 ml (in the submerged chamber) to ensure rapid drug equilibration. Drugs were removed by switching to the drug-free aCSF solution.

Extracellular field recordings were made in brain slices using standard techniques. The electrodes (thin-wall single-barrel borosilicate glass 1.2/0.9 mm outside diameter/inside diameter [OD/ID], World Precision Instruments) were pulled on a Sutter Instruments Flaming/Brown (P-87) micropipette puller and filled with 2 M NaCl. Electrode tips were broken to obtain resistances of 5–10 MΩ. Electrodes were positioned in the neocortex (layer V) with oblique fiber optic illumination. The extracellular signals were amplified 20×, and the low-pass Bessel filter (Corning Instruments 901) was set to a corner frequency of 1.05 kHz and visualized on a digital oscilloscope (Nicolet).

Amplitudes of 0 Mg^2^^+^-induced epileptiform discharges were determined by taking the average of the peak amplitude of the depolarizing envelope and the amplitude at the end of the afterdischarge. All values were presented as mean ± standard error of the mean (SEM). For each parameter the statistical differences were tested using the non-parametric Mann–Whitney *U*-test. Statistical significance was set at *p *< 0.05.****

## RESULTS

### SELECTIVE *IN VIVO* SEIZURE EXACERBATION WITH MK-801 in *STARGAZER* BUT NOT *TOTTERING *MICE

**FIGURE 1:**
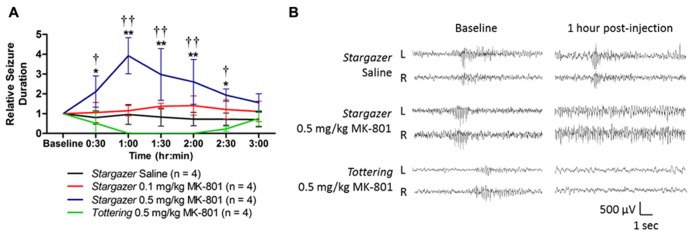
**(A)** Exacerbation of spontaneous spike-wave seizures following MK-801 in *stargazer *mice. Compared to ip saline, the total seizure duration is elevated by up to 448% 1 h post-injection with 0.5 mg/kg in *stargazer* mutants (*p < 0.05, **p < 0.0001). At the same dose, seizures are entirely suppressed in *tottering *mice (^†^*p *< 0.01, ^††^*p *< 0.0001 compared to *stargazer *0.5 mg/kg). A dose of 0.1 mg/kg was ineffective in *stargazer* mutant mice. Baseline seizure duration was obtained by summing burst durations over 30 min of acclimatized EEG recording. **(B)** Representative EEG traces at baseline and 1 h post-injection.

Within 15 min of intraperitoneal injection 0.5 mg/kg MK-801 dramatically prolonged spike-wave activity, increasing the duration of spiking by 448% at 1 h post-injection compared to saline (**Figure 1**, *p *< 0.0001). Electrographic spiking during these episodes slowed from 6–9 to ~3–4 Hz. A lower dose of 0.1 mg/kg MK-801 had no effect. To determine whether exacerbation by NMDA blockade was specific to the *stargazer *mutant, a second model of absence epilepsy, *tottering *mice, which bear a mutation in *Cacna1a *voltage gated**P/Q calcium channels, were injected with 0.5 mg/kg MK-801. In contrast to *stargazer*, a single dose of MK-801 eliminated spike-wave seizures in *tottering *within 1 h post-injection, which later returned to baseline levels over a 2 h period (**Figure 1**, *p *< 0.0001 compared to *stargazer *0.5 mg/kg).

### STARGAZIN IS SELECTIVELY EXPRESSED IN PV^+^ CELLS IN SOMATOSENSORY CORTEX

**FIGURE 2:**
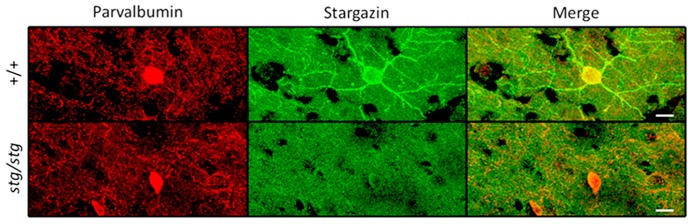
**Stargazin expression is restricted to PV^+^ interneurons in WT mouse somatosensory cortex (upper row).** Somatodendritic stargazin immunoreactivity is lost in PV^+^ interneurons in *stargazer *mouse (lower row). Co-labeling of stargazin and parvalbumin antibodies shown in layer 2/3 at 63× magnification (scale = 10 μm).

**FIGURE 3:**
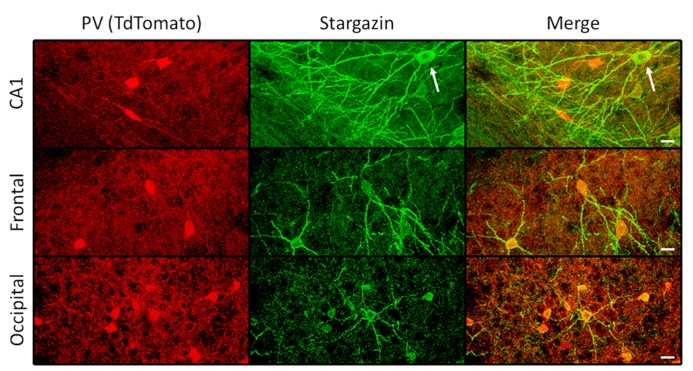
**Regional variation in stargazin immunoreactivity.** In the hippocampal CA1 region, both PV^+^ interneurons and pyramidal neurons (arrow) can express stargazin, whereas in frontal and occipital cortex, as in somatosensory regions, stargazin expression is limited to PV^+^/TdT^+^ interneurons (scale = 10 μm).

To explore the mechanism underlying paradoxical prolongation of seizures following NMDA receptor blockade, we investigated the cellular localization of stargazin in neocortex. Fluorescence immunohistochemistry with specific antibodies for stargazin labeled a subpopulation of neurons sparsely distributed throughout all cortical layers. Co-labeling with a specific antibody for parvalbumin (PV) revealed these were uniformly interneurons; in these PV^+^ cells, stargazin is highly expressed in the dendritic arbor and to a lesser degree, their soma (**Figure 2**, top). Of 71 PV^+^ interneurons identified through all layers of somatosensory cortex over a region of 1.6 × 10^-^^2^ cubic millimeters in an adult WT mouse, 68 (95.8%) expressed detectable stargazin immunoreactivity. There were no stargazin^+^ cells that were not also PV^+^. No stargazin staining could be detected in pyramidal neuronal processes in somatosensory cortex, unlike in the hippocampus, where staining in the apical dendrites of CA1 pyramidal neurons could be observed (**Figure 3**, top). In contrast, dendrites of neocortical PV^+^ cells in *stargazer *show no detectable stargazin staining, and only faint staining in the soma (**Figure 2**, bottom). To corroborate the PV^+^ co-localization, we also evaluated the cellular expression of stargazin using a floxed PV-Cre/Ai9 mouse expressing the red fluorescent protein tdTomato (TdT) in PV^+^ cells. In somatosensory cortex, 108 of 125 (86.4%) PV^+^ interneurons counted over a region of 1.9 × 10^-^^2^ cubic millimeters were immunopositive for stargazin. Again, no stargazin^+^ cells were detected that were not also TdT^+^. We noted six TdT^+^ cells in layers 5-6 which had a pyramidal morphology, as has been previously recognized in this PV-Cre line (Tanahira et al., 2009). None of these TdT^+^ pyramidal-shaped cells expressed stargazin; if excluded from the total, 108 of 119 (90.8%) TdT^+^ cells expressed stargazin.

To evaluate the extent of stargazin antibody specificity for PV^+^ interneurons throughout the cortex, other cortical regions were examined in an adult WT mouse expressing TdT in PV^+^ cells, sampling at least 6.4 × 10^-^^3 ^cubic millimeters for each area. These regions showed somewhat greater heterogeneity of stargazin co-expression with parvalbumin. In retrosplenial cortex, 40 of 71 (56%) TdT-labeled cells expressed stargazin in soma and dendrites. Similarly, 42 of 76 (55%) in entorhinal cortex; 16 of 46 (35%) in frontal cortex; and 20 of 59 (34%) in occipital cortex expressed somato-dendritic stargazin in TdT^+^ cells. All of the 226 stargazin^+^ cells sampled in somatosensory, entorhinal, frontal, and occipital cortex were TdT^+^ and hence PV^+^ (**Figure 3**, middle and bottom). In contrast, in CA1, piriform cortex, perirhinal cortex, and insular cortex, stargazin expression was found in both TdT-labeled and TdT-unlabeled cells (**Figure 3**, top).

### STARGAZIN IS LINKED TO GluA4 EXPRESSION IN CORTICAL INTERNEURONS AND LOSS IMPAIRS TRAFFICKING

**Figure 4:**
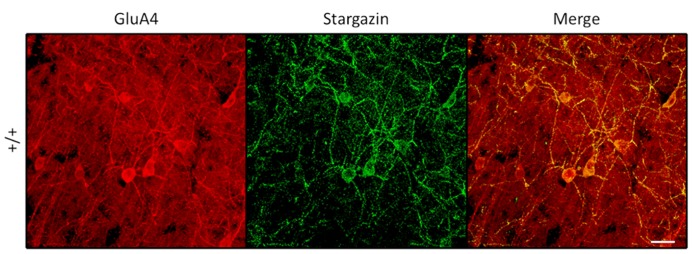
**Stargazin expression in PV^+^ interneurons, shown here in layers 5/6 ofWT somatosensory cortex, is somatodendritic and associated with dendritic GluA4 expression (scale = 20 μm)**.

**FIGURE 5:**
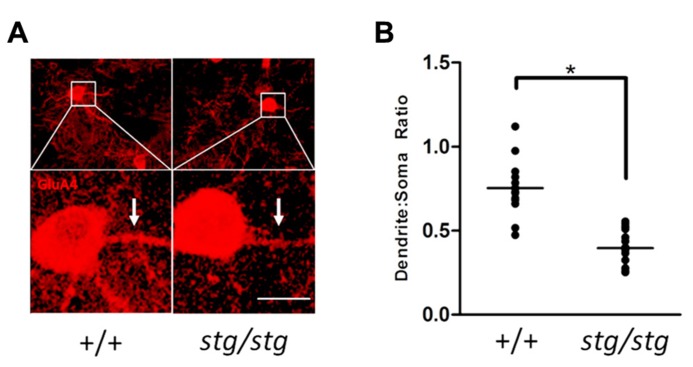
**(A)** Cortical PV^+^ interneurons in *stargazer* mutant mice show clear evidence of GluA4 mistrafficking in dendrites. Strong labeling in WT dendrites (arrows) is consistently diminished in *stargazer* interneurons. A relative decrease in the density of dendritic GluA4 immunoreactivity can be demonstrated by comparison of the dendrite:soma immunostaining ratio in *stargazer* interneurons compared to WT interneurons (scale = 10 μm). **(B)** Quantification of mean dendrite:soma ratio of GluA4 staining reveals a significant decrease in mutant PV^+^ interneurons (mean ratio of 0.753 for +/+, *n* = 15, vs 0.396 for *stg/stg*, *n* = 18, *p < 0.001).

Since PV^+^ interneurons express the calcium-permeable AMPA receptor subunit GluA4 (Chang et al., 2010), we next investigated the cellular co-localization of stargazin and GluA4 proteins in somatosensory cortex. Stargazin expression was associated exclusively with GluA4 expression in the soma and proximal dendrites of PV^+^ cells in all layers (**Figure 4**). Sampling 2.5 × 10^-^^2^ cubic millimeters of somatosensory cortex in an adult WT mouse revealed no stargazin^+^ cells (0/96) that were not also GluA4^+^. Since loss of stargazin protein reduces membrane insertion and stabilization of AMPA receptors, we searched for evidence that GluA4 trafficking was also impaired in *stargazer *PV^+^ cortical interneurons. PV^+^ cells that expressed GluA4 were readily identified in *stargazer s*omatosensory cortex, with clear staining of the soma; however, dendritic processes displayed visibly decreased staining for GluA4 in mutant PV^+^ cells compared to WT (**Figure 5A**). A total of 18 PV^+^ neurons from 4 *stg*/*stg* mice and 15 PV^+^ neurons from 3 +/+ mice met all of the inclusion and exclusion criteria for dendritic densitometric analysis. In PV^+^ interneurons, there was a significant, near twofold decrease in the dendrite to soma ratio of GluA4 staining in mutant compared to WT controls (52%, *p *< 0.001, **Figure 5B**).

### NMDA RECEPTOR ANTAGONISTS CPP AND MK-801 AUGMENT *STARGAZER* NEOCORTEX EXCITABILITY *IN VITRO*

**FIGURE 6:**
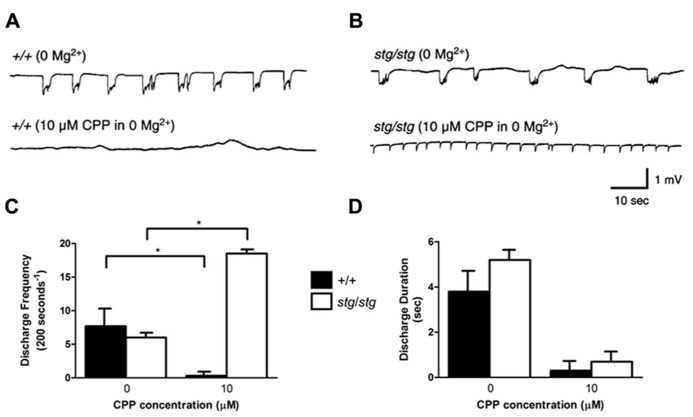
***Stargazer *mutant paradoxical cortical excitability defect is maintained *in vitro*.** **(A)** The frequency of spontaneous network discharges in WT cortical slices bathed in Mg^2^^+^-free solution (upper trace) is decreased in 10 μM CPP (lower trace). **(B)** In contrast, discharge activity in *stargazer *cortical networks is increased following CPP exposure. **(C)** Group data reveal mean discharge frequency in WT is significantly decreased by 10 μM CPP (*n* = 4) and increased in *stargazer* slices (*n* = 4), *p < 0.001. **(D)** No significant difference in mean discharge duration between WT and *stargazer* mice before or after administration of CPP.

In order to determine whether intracortical networks were sufficient to express the proepileptic effect of NMDA receptor antagonists, isolated cortical slices from WT and *stg/stg* mice were perfused with 0 Mg^2^^+^ aCSF to generate synchronous epileptiform discharges, which are due to NMDA receptor activation (Traub et al., 1994). Extracellular field recordings of layer V neurons revealed no significant differences between the two genotypes in the frequency of extracellularly recorded bursts before drug exposure. Application of the competitive NMDA receptor antagonist CPP (10 μM) nearly completely abolished the 0 Mg^2^^+^ epileptiform discharges in WT slices (number of discharges/200 s, 0 Mg^2^^+^: 7.7 ± 2.4, *n* = 4; 10 μM CPP: 0.3 ± 0.3, *n* = 4, *p *< 0.001, **Figures 6A,C**). However, application of 10 μM CPP elevated network excitability in the neocortex of *stargazer* mice by 308% (number of discharges/200 s, 0 Mg^2^^+^: 6.0 ± 0.4, *n* = 4; 10 μM CPP: 18.5 ± 0.3, *n* = 4, *p *< 0.001, **Figures 6B,C**). In both genotypes, there was a nearly equivalent and significant decrease in mean duration of burst discharges by 93 and 87% in WT and *stargazer* mice, respectively (duration of discharges, WT: 0 Mg^2^^+^: 3.8 ± 0.8 s, *n* = 4; 10 μM CPP: 0.3 ± 0.3 s, *n* = 4, *p *< 0.001; *stargazer*: 0 Mg^2^^+^: 5.2 ± 0.3 s, n = 4; 10 μM CPP: 0.7 ± 0.3 s, *n* = 4, *p *< 0.001, **Figure 6D**).

**FIGURE 7:**
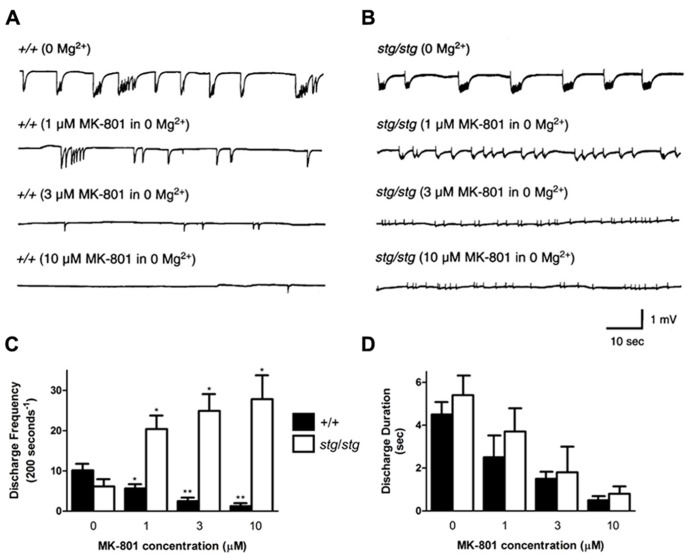
**Non-competitive NMDA receptor blockade with MK-801 also provokes paradoxical cortical network hyperexcitability *in vitro*.** **(A)** In 0 Mg^2^^+^, discharges in WT cortex show a dose-dependent reduction in duration and frequency with application of MK-801. **(B)** In contrast, spontaneous 0 Mg^2^^+^-induced network bursting in *stargazer *cortical slices accelerates with increasing doses of MK-801. **(C)** Graphical representation of divergent response to increasing doses of MK-801 in WT and *stargazer *mice (*p < 0.05, **p < 0.005). In **(D)**, there is a coordinate reduction in discharge duration with no significant difference between groups at each incremental concentration of MK-801.

Likewise, the non-competitive NMDA receptor antagonist MK-801 was perfused at varying concentrations on WT neocortical slices, where it nearly abolished 0 Mg^2^^+^-induced discharge activity at higher doses (**Figure 7A**). Bath application of MK-801 in 0 Mg^2^^+^ aCSF in WT slices decreased the mean discharge frequency by 45% at 1 μM, 75% at 3 μM, and 88% at 10 μM (number of discharges/200 s, 0 Mg^2^^+^: 10.2 ± 1.4, *n* = 4; 1 μM MK-801: 5.7 ± 0.8, *n* = 4, *p *= 0.0273; 3 μM MK-801: 2.5 ± 0.5, *n* = 4, *p *= 0.0019; 10 μM MK-801: 1.3 ± 0.3, *n* = 4, *p *= 0.0007, **Figure 7C**). The discharge frequency of the 0 Mg^2^^+^-induced bursts never increased at any point during application of MK-801. In addition, application of MK-801 to WT slices decreased the mean discharge duration by 44% at 1 μM, 66% at 3 μM, and 89% at 10 μM (0 Mg^2^^+^, 4.5 ± 0.6 s, *n* = 4; 1 μM MK-801, 2.5 ± 1.1 s, *n* = 4, *p *> 0.05; 3 μM MK-801: 1.5 ± 0.3 s, *n* = 4, *p *= 0.0045; 10 μM MK-801: 0.5 ± 0.1 s, *n* = 4, *p *= 0.0005, **Figure 7D**). In contrast, application of 1, 3, and 10 μM MK-801 to *stargazer* slices did not block 0 Mg^2^^+^-induced bursting (**Figure 7B**), but rather, similar to the effects of CPP, increased the mean frequency of network discharges by 331% at 1 μM, 405% at 3 μM, and 451% at 10 μM (number of discharges/200 s, 0 Mg^2^^+^: 6.2 ± 1.4, *n* = 4; 1 μM MK-801: 20.4 ± 3.9, *n* = 4, *p *= 0.0139; 3 μM MK-801: 24.9 ± 4.3, *n* = 4, *p *= 0.0061; 10 μM MK-801: 27.8 ± 5.8, *n* = 4, *p *= 0.0108, **Figure 7C**). MK-801 decreased the mean duration of the discharges by 32% at 1 μM, 68% at 3 μM, and 85% at 10 μM (0 Mg^2^^+^, 5.4 ± 0.9 s, *n* = 4; 1 μM MK-801, 3.7 ± 1.1 s, *n* = 4, *p *> 0.05; 3 μM MK-801: 1.8 ± 1.3 s, *n* = 4, *p *= 0.0156; 10 μM MK-801: 0.8 ± 0.5 s, *n* = 4, *p *= 0.0023, **Figure 7D**). These data demonstrate that blockade of NMDA receptors with both competitive and non-competitive antagonists paradoxically increases neocortical excitability in *stargazer* cortex without the participation of subcortical circuitry.

### NMDA RECEPTOR-DEPENDENT CORTICAL HYPEREXCITABILITY IS MEDIATED BY INHIBITORY INTERNEURONS

**FIGURE 8:**
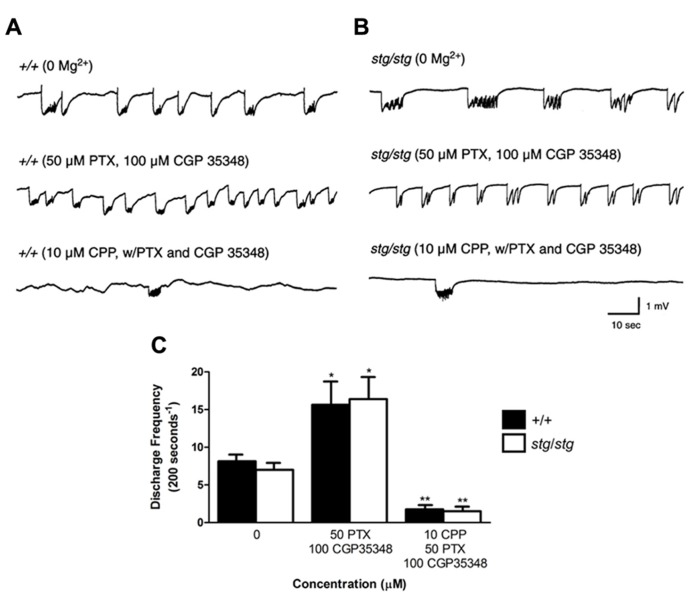
**Paradoxical exacerbation in *stargazer* cortex requires GABAergic synaptic transmission.** **(A,B)** Spontaneous 0 Mg^2^^+^-induced network discharges in WT and *stargazer *cortex (upper traces) are accelerated by combined GABA_ A_ and GABA_ B_ receptor blockade (middle traces); however, in this disinhibited condition, application of 10 μM CPP strongly and equally attenuates network bursting in both genotypes (lower traces). **(C)** Exacerbation of epileptiform discharges by CPP is not evident in WT or *stargazer *slices with complete GABAergic blockade (*n* = 4 per group, *p < 0.05, **p < 0.001).

In order to examine the hypothesis that NMDA receptor antagonism mediates its paradoxical excitatory effect in *stargazer* neocortex through inhibitory interneurons, we blocked GABAergic transmission within the neocortical circuit with GABA receptor antagonists and re-examined the effects of CPP on the 0 Mg^2^^+^-induced discharges in the fully disinhibited circuit. After co-application of 50 μM PTX, a GABA_ A_ receptor antagonist, and 100 μM CGP35348, a GABA_ B_ receptor antagonist, synchronous epileptiform discharges elicited in 0 Mg^2^^+^ aCSF increased in frequency in both WT and *stargazer* mice (number of discharges/200 s in WT, 0 Mg^2^^+^: 8.2 ± 0.7, *n* = 4; 50 μM PTX and 100 μM CGP35348: 15.7 ± 2.9, *n* = 4, *p *= 0.0450; in *stargazer, *0 Mg^2^^+^: 7.0 ± 0.8, *n* = 4; 50 μM PTX and 100 μM CGP35348: 16.4 ± 2.9, *n* = 4, *p *= 0.0392, **Figure 8**). However, perfusing 10 μM CPP in the presence of the two antagonists significantly reduced epileptiform activity, but to a similar extent in both genotypes (number of discharges/200 s, WT: 1.8 ± 0.4, *n* = 4; *stargazer*: 1.5 ± 0.3, *n* = 4, *p *< 0.001, **Figure 8**). These findings support the hypothesis that the paradoxical response to NMDA receptor antagonism in *stargazer *mice is mediated by cortical GABAergic interneurons.

## DISCUSSION

Our findings pinpoint a likely synaptic mechanism for paradoxical spike-wave seizure exacerbation due to NMDA receptor antagonism, showing that it is present in *stargazer*, but not *tottering* mutant mice, and thus depends upon an interneuron-specific AMPA receptor dendritic trafficking abnormality. We show that expression of stargazin in adult mouse somatosensory cortex is confined to a singular population of fast-spiking, PV^+^ interneurons. These cells are primarily responsible for fast synaptic inhibition of neurons in both superficial and deep pyramidal cell layers (DeFelipe, 1997). This specificity was most pronounced in the somatosensory cortex, a region proposed to possess a low threshold for involvement in aberrant thalamocortical oscillations in rodent models (Polack et al., 2007). In *stargazer* mice, we identified a concomitant deficit in dendritic AMPA receptor trafficking in these interneurons. *In vitro* cortical network hyperexcitability, as evidenced by epileptiform bursting in a magnesium-free environment, was enhanced by NMDA receptor antagonists in *stargazer* somatosensory cortex but reduced in WT mice. The paradoxical excitability difference between epileptic and non-epileptic cortex in response to CPP was no longer evident when GABA receptors were completely blocked, indicating that hyperexcitability induced by NMDA receptor antagonism was mediated through interneurons.

### PARADOXICAL SEIZURE EXACERBATION WITH MK-801 IS SPECIFIC TO *STARGAZER* MICE

*In vivo* administration of the NMDA receptor antagonist MK-801 markedly exacerbated seizures in *stargazer *mice but had an opposite effect in *tottering *mice, indicating that the paradoxical seizure aggravation is gene-linked, rather than a non-specific modulation of spike-wave seizures due to a confounding variable such as sedation. The excitatory effect of MK-801 was similar to that of CPP in *stargazer *mice (Nahm and Noebels, 1998), confirming that both competitive (CPP) and non-competitive NMDA (MK-801) receptor antagonism, rather than an off target effect of either drug, mediated the paradoxical response. MK-801 has also been shown to reduce spike-wave seizures in other rodent models of epilepsy, including the kindling model (Lojková et al., 2006) as well as the WAG/Rij (Wistar Albino Glaxo from Rijswijk; Peeters et al., 1989; Citraro et al., 2006) and Genetic Absence Epilepsy Rats from Strasbourg (GAERS) models of absence epilepsy (Koerner et al., 1996), further supporting the specific association of the paradoxical response to NMDA receptor antagonism with the *stargazer* trafficking defect. In a prior study of *stargazer* mice, CPP exacerbation of spike wave activity was also clearly observed, although no significant difference in total seizure duration over the time period measured was reported (Aizawa et al., 1997). In that study, MK-801 at a dose of 0.5 mg/kg did not stimulate discharge activity, but did produce an “irregular pattern” of EEG epileptiform activation that could represent a prolonged period of absence status epilepticus as we have occasionally observed.

### STARGAZIN EXPRESSION IS REGIONAL AND LIMITED TO FAST-SPIKING INTERNEURONS IN SOMATOSENSORY CORTEX

The specificity of stargazin expression in PV^+^ interneurons of the somatosensory cortex initially seemed at variance with previous findings that its phosphorylation is dependent on CaMKII (calcium/calmodulin-dependent protein kinase II), an enzyme which is restricted to excitatory neurons (Tomita et al., 2005; Tsui and Malenka, 2006; Opazo et al., 2010). However, these studies investigated stargazin function in the hippocampus, not in the cortex. The lack of stargazin expression in excitatory cells within somatosensory cortex, however, is consistent with the finding that phosphorylation of stargazin is dependent on CaMKII in dissociated hippocampal, but not cortical, cultured neurons (Inamura et al., 2006). In addition, a recent study found that stargazin is specifically expressed in interneurons in primary dissociated cortical cell cultures (Tao et al., 2013). Finally, the relative specificity for stargazin in inhibitory neurons rather than principal cells is a pattern that recurs in the cerebellum (Shevtsova and Leitch, 2012) and the thalamus (Menuz and Nicoll, 2008).

### COMPARTMENTAL AMPA TRAFFICKING DEFECT IN *STARGAZER* INTERNEURONS

In the absence of functional stargazin, GluA4 trafficking may still be partially compensated by TARP redundancy mediated by other co-expressed gamma subunits (Menuz et al., 2008) as well as the recently described “TARPless” expression of calcium-permeable surface AMPA subunits at some synapses (Bats et al., 2012). Several other candidate interacting molecules for AMPAR trafficking have been proposed (Jackson and Nicoll, 2011). However, the relatively decreased dendrite to soma ratio of GluA4 expression in *stargazer* PV^+^ interneurons indicates that these potential compensatory mechanisms are incomplete and therefore define a specific vulnerability in this cellular subgroup. When specific antibodies for remaining gamma subunit family members become available, the exact cellular TARP expression profiles may clarify the cellular populations at risk for AMPA receptor impairment due to *Cacng *subunit mutations. 

The exclusive association of stargazin with GluA4^+^ neurons in the neocortex is phenotypically consistent with the recent finding that the *Gria4* knockout mouse, which is devoid of GluA4 expression, also displays similar spike-wave seizures (Beyer et al., 2008; Paz et al., 2011). In both *stargazer* and *Gria4* knockout mice, there is evidence for thalamic disinhibition due to a specific deficit of synaptic excitation at fast-spiking PV^+^ inhibitory neurons in the RTN (Barad et al., 2012). We therefore posit the presence of a similar functional defect of intracortical inhibition in this model due to a parallel deficit in the ability to excite fast-spiking PV^+^ interneurons in the somatosensory cortex.

### DOES DEFICIENT DENDRITIC GluA4 TRAFFICKING IMPAIR SYNAPTIC ACTIVATION OF INTERNEURONS IN *STARGAZER *CORTEX?

The degree to which abnormal dendritic GluA4 distribution alters synaptic activation of interneurons and hence the efficiency of GABA release has not been examined in this study, and functional recordings from PV^+^ cells activated by feedforward excitatory synapses will be required to determine whether AMPA receptor mistrafficking reduces the strength of this input. One impediment to studying this lies in the coincidental proximity of the stargazin locus on chromosome 15 to the parvalbumin locus, separated by only 0.01 cM. This complicates the ability to identify these cells for physiological study in *stargazer *cortex using a simple genetic cross with parvalbumin promoter-driven reporters. Future studies using defined presynaptic glutamate activation and *post hoc* immunochemical identification of PV^+^ cells will be required to support this hypothesis. However, it is worth noting that the impairment of synaptic excitatory activation in hippocampal neurons depleted of the homologous TARP gamma-8 subunit, which constitutes the majority of TARP-mediated AMPA receptor surface expression in these cells, was small (Rouach et al., 2005).

### INTERNEURON-DEPENDENT NETWORK HYPEREXCITABILITY DUE TO NMDA RECEPTOR ANTAGONISM IN *STARGAZER* MICE

Since we observed a reduction of dendritic AMPA receptor density in parvalbumin^+^ interneurons that are critical for feedforward inhibition, and because stargazin does not directly traffic NMDA receptors (Chen et al., 2000), we reasoned that the synaptic activation of these inhibitory neurons would largely depend on NMDA receptors. Enhanced NMDA receptor-mediated excitation of inhibitory neurons of the RTN has been reported in *stargazer* mice (Lacey et al., 2012), but the cortical node of the thalamocortical loop has not previously been investigated. This hypothesis was tested in a magnesium-free environment, which is a useful medium for evaluating the effect of anti-epileptic drugs (Aram and Lodge, 1988). Magnesium normally blocks the activation of NMDA receptors, and its omission from the *in vitro* bathing solution results in spontaneous burst discharges (Traub et al., 1994). Although 0 Mg^2^^+^-induced discharges do not have the same pathophysiology as *in vivo* spike and wave cortical discharges, they provide a reliable functional measure of cortical circuit excitability. Specifically, these discharges arise as a consequence of saturation of fast inhibition, which can be overcome with incremental NMDA receptor antagonism (Benardo, 1993). In solutions containing 0 Mg^2^^+^ and CPP, fast inhibition relies on AMPA receptor-mediated activation of interneurons (Ling and Benardo, 1995), which we predicted to be compromised in *stargazer *mice. This effect is consistent with our results showing a reduced spontaneous burst frequency with CPP and increasing doses of MK-801 in WT slices in a magnesium-free environment. Accordingly, both competitive and non-competitive blockade of NMDA receptors caused a paradoxical increase in discharges in *stargazer* cortex, consistent with a compromise of fast inhibition. The significant paradoxical increase in discharge rate supports the hypothesis that when AMPA receptor-mediated transmission is impaired, NMDA receptor antagonism in 0 Mg^2^^+^ not only fails to suppress seizure activity, but rather causes a further, dose-dependent reduction in inhibition. After pharmacologically removing GABAergic inhibitory inputs of the *stargazer* network with picrotoxin and CGP35348, CPP application no longer produced a paradoxical excitability increase, indicating that the excitatory effect of CPP in *stargazer *mice is indeed mediated through inhibitory neurons within the isolated cortical network. Furthermore, GABA receptor antagonists applied independently of CPP significantly increased the frequency of the 0 Mg^2^^+^ discharges in neocortex of both *stargazer* and WT mice, which itself demonstrates that disinhibition can increase the frequency of 0 Mg^2^^+^ discharges in the cortex and is consistent with the interpretation that CPP mediates its excitation in the mutant by aberrant disinhibition.

Our findings in the isolated neocortex point to potential regional differences in the role of NMDA receptors and network oscillations at various nodes of the thalamocortical loop. Interestingly, in thalamic slices of *stargazer* mice bathed in 0.5 mM Mg^2^^+^, the application of 50 uM APV (DL-2-amino-5-phosphonovalerate), a competitive NMDA receptor antagonist like CPP, caused a significant decrease in evoked oscillations in thalamic nuclei, similar to WT slices (Lacey et al., 2012). Thus, the paradoxical effect of seizure exacerbation observed *in vivo* may principally reside in the low threshold initiation zones in the somatosensory cortex. These data therefore support a more general hypothesis that a reduction in the strength of cortical inhibitory interneurons provides an attractive candidate mechanism for paradoxical seizure exacerbation by anti-epileptic drugs in some patients with absence epilepsy.

## Conflict of Interest Statement

The authors declare that the research was conducted in the absence of any commercial or financial relationships that could be construed as a potential conflict of interest.
